# Respire to ripe: The involvement of the mitochondrial alternative oxidases in tomato fruit ripening

**DOI:** 10.1093/plphys/kiaf454

**Published:** 2025-09-27

**Authors:** Pablo Ignacio Calzadilla, Paula Muñoz

**Affiliations:** Assistant Features Editor, Plant Physiology, American Society of Plant Biologists; Institute for Integrative Biology of the Cell (I2BC), Université Paris-Saclay, CEA, CNRS, Gif-sur-Yvette cedex 91198, France; Department of Earth and Environmental Sciences, Faculty of Science and Engineering, University of Manchester, Manchester M13 9PT, United Kingdom; Department of Earth and Environmental Sciences, Faculty of Science and Engineering, University of Manchester, Manchester M13 9PT, United Kingdom

Respiration is the fundamental process in which cells break down sugars in the presence of oxygen to produce energy in the form of ATP, which fuels cellular maintenance, metabolic reactions, and many physiologic processes of living organisms. In plants, respiration is not only relevant for growth, development, and stress responses but becomes particularly important in highly energy-demanding processes such as fruit ripening (Jiang et al. 2007). During ripening, respiration provides the metabolic activity necessary for synthesizing the protein, hormones, and pigments that will give rise to the mature fruit. In tomato (*Solanum lycopersicum*), fruit ripening is accompanied by chlorophyll degradation, ethylene production, and the progressive accumulation of carotenoid pigments (Su et al. 2015). Although the participation of respiration in climacteric fruit ripening is well documented, its in vivo regulation and function at different ripening stages remain unclear.

Plant cellular respiration involves glycolysis in the cytosol, the tricarboxylic acid cycle in the mitochondrial matrix, and electron transport in the inner mitochondrial membrane (Fernie et al. 2004). Within the electron transport reactions, several respiratory pathways exist. The main route leading to ATP production involves different respiratory complexes, ubiquinone, and the cytochrome *c* oxidase (COX). However, alternative respiratory pathways allow mitochondrial redox balance at the expense of ATP generation (Vanlerberghe 2013). In particular, the alternative oxidase (AOX) oxidizes ubiquinone and reduces O_2_ to H_2_O, thereby bypassing the proton pumping of complexes III and IV (Del-Saz et al. 2018). Several AOX genes have been reported in tomato and are suggested to have a role in climacteric respiration and fruit ripening (Xu et al. 2012). Moreover, a plastid terminal oxidase (PTOX) catalyzes a similar reaction in the chromoplast (a specialized plastid in fruits), which can produce ATP with the NAD(P)H dehydrogenase complex in a process called *chromorespiration* (Grabsztunowicz et al. 2019). This process has been demonstrated to generate ATP during late ripening stages in tomato fruits (Renato et al. 2014). However, the coordinated regulation of the different respiratory pathways during fruit ripening remains unknown.

In this issue of *Plant Physiology*, Iglesias-Sanchez et al. (2025) determined the in vivo activities of COX and AOX during ripening in tomato and their impact on the expression of ripening-related genes and primary and carotenoid metabolism. The authors used the Ailsa Craig wild type tomato line and the PTOX-defective *ghost* mutant (Barr et al. 2004) to disentangle the role of PTOX during ripening. They found that respiration increased at the breaker ripening stage when ethylene increased. Measurements were performed by using an oxygen electrode and ^18^O discrimination, a technique to assess oxygen isotope fractionation between alternative respiratory pathways. ^18^O discrimination by the AOX and COX pathways was determined in the presence of the chemical inhibitors KCN and SHAM, respectively. In vivo AOX activity (v_alt_) was the main contributor to climacteric respiration. By contrast, in vivo COX activity (v_cyt_) was detected only until the breaker ripening stage, while chromorespiration gains significance at the end of ripening.

Due to the importance of AOX activity during tomato fruit ripening, Iglesias-Sanchez and colleagues decided to study and knock out the AOX genes responsible for this activity. The expression of *AOX1a* and *AOX1b* was quantified. While *AOX1b* expression did not change significantly during ripening, *AOX1a* expression steadily increased until the end of this process. Thus, the authors generated 2 homozygous *aox1a* mutant lines through CRISPR/Cas9 and analyzed their fruit-ripening process. The knockout mutants were able to reproduce and develop fruits. However, fruit weight, size, and number were lower than in wild type plants. Moreover, a delay was observed in the first fruit development and the onset of ripening, mainly due to an increase in the transition times to and from the breaker ripening stage.

To further characterize the relevance of AOX in fruit ripening, Iglesias-Sanchez et al. measured total respiration and AOX capacity in the *aox1a* mutants. Impairments in climacteric respiration were observed in the 2 mutant lines, in agreement with the delay in the onset of ripening. The expression of ethylene-responsive genes was also delayed or decreased, supporting the involvement of AOX respiration in the ethylene-induced response. Finally, metabolic profiling showed alterations in primary metabolism and carotenoid contents in the *aox1a* mutant lines. Interestingly, aspartate and methionine accumulation (ethylene synthesis precursors) was affected in the absence of *AOX1a*, suggesting a more direct link between AOX respiration and ethylene response.

In summary, Iglesias-Sanchez et al. (2025) characterize the respiratory pathways involved in fruit ripening in tomato, highlighting the importance of the AOX in climacteric respiration (Fig.). Through analysis of the *ghost* tomato line and the generation of *aox1a* mutants by CRISPR/Cas9, the authors concluded that COX activity and chromorespiration are mainly relevant during the early and late stages of tomato fruit ripening, respectively. In contrast, AOX activity triggers high respiratory fluxes, providing precursors for carotenoid synthesis and the production of key metabolites (i.e. aspartate and methionine) for ethylene in tomato. The work provides a better understanding of the metabolic and energy fluxes driving ripening in this economically relevant fruit.

**Figure.**
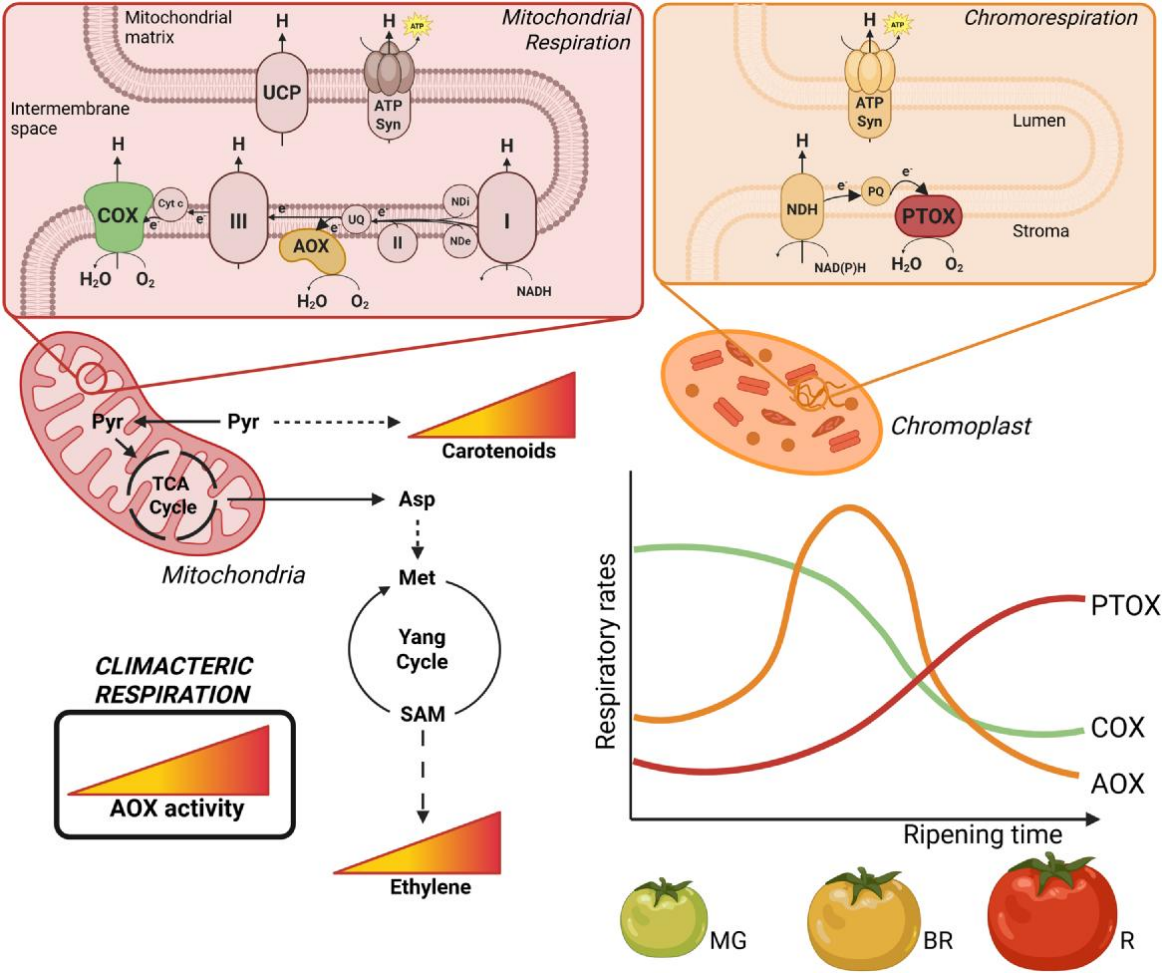
Mitochondrial AOX is the main contributor to climacteric respiration in tomato. Several respiratory pathways participate in fruit ripening in tomato, becoming significant at different stages of this process. While COX and PTOX activity contributes to early and late ripening, respectively, AOX triggers a respiratory burst at the breaker stage (BR). The latter is the main contributor to climacteric respiration, providing key precursors for carotenoid and ethylene synthesis during tomato fruit ripening. Asp, aspartate; ATP Syn, ATP synthase; Cyt c, cytochrome *c*; Met, methionine; MG, mature green stage; NDH, plastidial NADH dehydrogenase complex; NDi and NDe, internal and external type II dehydrogenases; PQ, plastoquinone; Pyr, pyruvate; R, red stage; SAM, S-adenosylmethionine; UCP, uncoupling protein; UQ, ubiquinone. Created in BioRender. https://BioRender.com/8i1djcq.
